# Timing of enteral nutrition initiation in septic shock patients: only the gut knows

**DOI:** 10.1097/JS9.0000000000002424

**Published:** 2025-04-29

**Authors:** Qinjie Liu, Jie Wu

**Affiliations:** a Department of General Surgery, Zhongda Hospital, School of Medicine, Southeast University, Nanjing, China; b Department of Critical Care Medicine, Jiangsu Provincial Key Laboratory of Critical Care Medicine, Zhongda Hospital, School of Medicine, Southeast University, Nanjing, Jiangsu, China

*To the Editor,*

We read the article by Xu *et al* about abdominal perfusion pressure (APP) as a critical predictor of survival in patients with intra-abdominal hypertension (IAH) with great interest^[1]^. Their research yields substantial implications that extend beyond the prediction of mortality, particularly in the context of making decisions regarding nutritional support for patients with septic shock.

Currently, the guidelines for starting enteral nutrition (EN) in septic shock mainly depend on assessing hemodynamic stability^[2]^. Usually, we use the dosage of vasopressors as the assessment method. The European Society for Clinical Nutrition and Metabolism (ESPEN) suggested being careful with EN when high-dose vasopressors are needed or in patients with intra-abdominal hypertension without abdominal compartment syndrome, whereas temporary reduction or discontinuation of EN should be considered when intra-abdominal pressure values further increase under EN^[2]^; Notably, Murphy et al. found that the prevalence of IAH in intensive care units (ICUs) is much higher than what was thought before^[3]^. Therefore, the neglect of intra-abdominal pressure levels in ICU patients may cause many of them to either miss beneficial nutritional opportunities or face inappropriate risks.

Furthermore, the relationship between the dosage of vasopressors and mesenteric perfusion is non-linear. Krejci et.al demonstrated that phenylephrine can keep mesenteric perfusion normal within certain dosage ranges, but norepinephrine and epinephrine can greatly reduce the blood flow in the intestines^[4]^. Moreover, inconsistent definitions of “hemodynamic stability” across centers affect care standardization. These limitations highlight the need for direct intestinal status assessment in septic shock patients.

Sepsis causes complex changes in the intestine’s physiology, including wall edema, shortening, and intraluminal gas accumulation, all of these changes directly reflected in intra-abdominal pressure (IAP)^[5]^. Furthermore, APP, calculated as mean arterial pressure minus IAP, provides a more comprehensive reflection of intestinal perfusion status. When APP <60 mmHg, it is related to organ dysfunction. Xu and his colleagues’ research^[1]^ shows that APP is better than just measuring IAP or MAP when it comes to predicting the results for patients with IAH. Applied to EN decisions, this suggests that even with mildly elevated IAP, patients with preserved APP (>60-70 mmHg) might safely receive low-dose EN, while those with normal IAP but significantly reduced APP may require more cautious.

Recent technological advancements have made this approach more feasible. The novel continuous bladder pressure monitor can capture of immediate responses following EN initiation and identification of transient but significant pressure fluctuations. Through continuous monitoring, clinicians may identify specific “intolerance patterns” such as sustained increases or high-amplitude fluctuations. This is valuable information. It can guide real-time adjustments of the EN rate.

We suggest an integrated clinical paradigm. First, assess the baseline IAP/APP before starting EN and evaluate whether EN can be used. Then, start EN at a low rate (10–20 mL/h) and regularly monitor the IAP/APP; Third, make dynamic adjustments according to the observed response patterns. Fourth, continuous optimization as patient condition improves. This physiologically-based approach represents a fundamental shift from static thresholds to individualized nutritional therapy.

This paradigm might be particularly beneficial for specific patient subgroups who are at a high risk of IAH, such as the patients with severe fluid overload, received abdominal surgery, obesity, undergoing mechanical ventilation. But there are still some challenges during implementation, including standardization of measurement techniques and interpretation of dynamic changes. We suggest that multidisciplinary protocols incorporating both Intensive care physician and nutrition specialists could facilitate adoption. Decision support tools might help make things more consistent among providers and institutions. Shifting to this way of doing things needs only a little extra resource. But it has a lot of potential to improve nutritional support and reduce complications.

There are still some important knowledge gaps. These include standardization of EN-specific IAP/APP response patterns, establishment of population-specific thresholds, and integration with complementary assessment methods. What’s more, outcome studies comparing this approach against standard practice are urgently needed.

In summary, the work by Xu *et al*^[1]^ provides a critical foundation for this paradigm shift. We believe that IAP/APP monitoring offers the potential to transform nutritional support in septic shock by replacing arbitrary thresholds with individualized, physiology-driven decisions that could improve clinical outcomes for these vulnerable patients.

## Data Availability

Data sharing is not applicable to this article.
